# Access to an outdoor open pack promotes estrus activity in dairy cows

**DOI:** 10.1371/journal.pone.0308182

**Published:** 2024-08-08

**Authors:** Anne-Marieke C. Smid, Tracy A. Burnett, Augusto M. L. Madureira, Kathryn J. McLellan, Claire S. Wegner, Marina A. G. von Keyserlingk, Daniel M. Weary

**Affiliations:** 1 Faculty of Land and Food Systems, Animal Welfare Program, The University of British Columbia, Vancouver, British Columbia, Canada; 2 Faculty of Veterinary Medicine, The University of Calgary, Calgary, Alberta, Canada; 3 Faculty of Land and Food Systems, The University of British Columbia, Vancouver, British Columbia, Canada; 4 University of Guelph, Ridgetown, Ontario, Canada; Sathyabama Institute of Science and Technology, INDIA

## Abstract

Dairy cows have a partial preference to access an outdoor deep-bedded pack, but the effects of continuous access to an outdoor area on estrous behaviors has not been studied. Our objective was to investigate if access to an outdoor open deep-bedded pack improves the expression of estrus behaviors. We enrolled 60 lactating Holstein cows directly after calving and followed them each for 12 weeks. Cows were housed in a single freestall pen holding 36 cows at a time, with a dynamic group composition. Half of the cows were randomly assigned to the OUTDOOR treatment; these cows had access to an outdoor open pack via an automated selection gate. INDOOR cows were housed together with OUTDOOR cows but were not allowed outdoor access. All cows were fitted with an automated activity monitor (AAM) 21 ± 3 d before expected calving date. Estrous behaviors (i.e., standing to be mounted and mounting behaviors) were continuously monitored using video, and the intensity of mounting (i.e., the number of standing to be mounted and other mounting behaviors per hour) was calculated per estrus event per cow. Temperature and humidity were monitored by data loggers indoors, and these data were used to calculate the Temperature Humidity Index (THI). Following an alert from the AAM, cows were checked to detect the presence of a dominant preovulatory follicle and an absence of a mature corpus luteum (CL) by rectal ultrasonography following milking, as well as 7 d thereafter to confirm ovulation by the presence of a new CL. A total of 94 estrus events were used in the final analysis. INDOOR cows tended to have a lower mounting intensity with increasing indoor THI, but no such effect was noted for OUTDOOR cows. The total number of cows in estrus positively influenced mounting intensity. OUTDOOR cows had a longer duration of estrus, as measured by the automated monitors, compared with INDOOR cows (12.4 ± 0.7 h versus 9.9 ± 0.8 h). Estrus event number and the total number of cows in estrus were both positively associated with estrus duration. These results indicate that access to an outdoor pack tended to increase mounting behavior for freestall housed dairy cows, especially during periods of elevated THI. We conclude that providing dairy cows access to an outdoor area can help with estrus detection and may thus help improve the reproductive programs on dairy farms.

## Introduction

The primary sign of estrus is when cows stand immobile to be mounted [1–3]. Secondary signs include mounting behavior and chin resting [3]. Detecting estrus can be challenging, in part due to the shorter duration and lower intensity of estrous expression in high-producing dairy cows [4]. Large herd sizes [5] and suboptimal housing conditions [1] can also make visual estrus detection more difficult. A questionnaire by Denis-Robichaud et al. [6] reported that the success of reproductive programs was considered a key challenge by producers, and that cow housing characteristics—including flooring and space availability—hindered their ability to detect estrus.

Pasture access can improve expression of estrus. For instance, cows on pasture stand to be mounted more often than cows kept in a freestall barn [7, 8], possibly explained by the type of flooring provided indoors. Other work indicated that the duration of estrus was lower in cows observed on concrete flooring compared with those on dirt surfaces [9]. Concrete flooring can inhibit natural locomotor behavior [10], especially if the flooring is wet and slippery [11], which may make cows more hesitant to mount. Palmer et al. [8] reported that freestall housed cows had more slips and falls when attempting to mount, compared with cows on pasture. Similarly, Boyle et al. [12] observed that heifers out-wintered on an outdoor wood-chip pad had overall fewer slips, trips and falls compared with heifers housed in a freestall barn. When cows were able to choose between mounting a cow on concrete or dirt, they were 3 times more likely to mount on dirt [13].

Our aim was to test the influence of free access to an outdoor open wood-chip pack (i.e., a fenced, outdoor area, also called a ‘paddock’) on estrous behaviors in freestall-housed dairy cows. We hypothesized that cows with access to the outdoor pack would perform more mounting and standing to be mounted behaviours than cows without access.

## Materials and methods

### Cows and treatment

This experiment took place between July 2017 and February 2018 at The University of British Columbia’s (UBC) Dairy Education and Research Centre (Agassiz, BC, Canada). The UBC Animal Care Committee approved the experiment and all procedures (Protocols A15–0082 and A14–0290).

A total of 60 lactating Holstein cows were enrolled in this study. Cows were assigned to either the OUTDOOR or INDOOR treatment: OUTDOOR cows were housed in a freestall pen and allowed free access to an outdoor wood-chip pack via an automatic selection gate (Lely Grazeway^™^); INDOOR (i.e., control) cows were housed in the same freestall pen (hereafter referred to as the experimental pen) but did not have access to the outdoor pack. The first cow was assigned to the INDOOR treatment; thereafter cows were assigned alternately to counterbalance any effect of calving date, with occasional reversals to balance for parity (OUTDOOR: 2.7 ± 1.6 lactations; INDOOR: 2.5 ± 1.3 lactations; mean ± SD), if assistance was required at calving (OUTDOOR: 5 cows; INDOOR: 4 cows) and if cows had previous experience with an outdoor open pack (OUTDOOR: 15; INDOOR: 13). Primiparous (1^st^ lactation) and multiparous (2^nd^ or greater lactations) cows were treated separately in the process of balancing these variables. Multiparous cows were also balanced for previous 305-d mature equivalent milk yield (305ME: OUTDOOR: 12,423 ± 2,222 kg; INDOOR: 12,723 ± 2,004 kg).

Cows diagnosed with any health condition, including lameness (score 4 or 5; [14]) at the time of calving were not included in the experiment; these exclusion criteria were determined *a priori*. Personnel who performed the rectal palpations were blind to the treatment; the two observers who conducted the video analyses were aware of the treatment due to the nature of this experiment.

Cows were defined as cystic or cycling irregularly when they had compromised ovarian patterns (i.e., multiple estrus events within a short period of time without ovulation) and/or a large persistent follicle (>20 mm) lasting for at least 7 days without ovulation [15, 16].

Experimental cows were kept in the experimental pen for 84 ± 1 d (range: 83–89 d), after which no data were collected.

### Housing, management and diet

**Fig 1:**
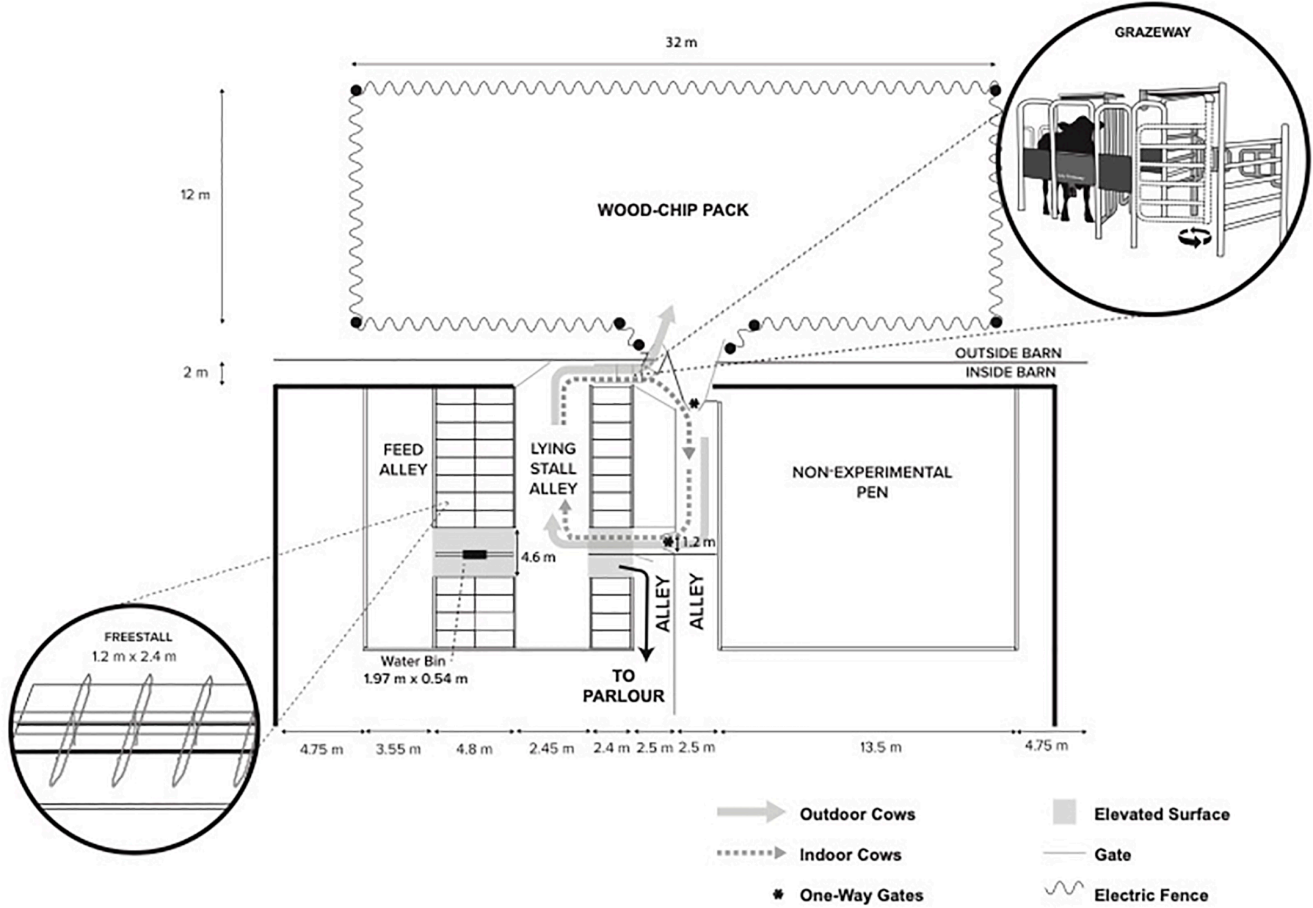
Schematic of experimental areas used.

Cows were housed in a mechanically ventilated wood-framed freestall barn (42 x 93 m; Fig 1) with a north-south orientation and curtained sidewalls. The experimental pen consisted of 36 lying stalls, configured in 3 rows of 12 stalls with a cross-over. Stalls were filled with approximately 40 cm of washed river sand, replenished every other week, and cleaned twice daily during milking time. Stalls were divided by Dutch-style partitions spaced 1.2 m wide center-to-center with the neck rail placed 1.4 m above the stall surface and 1.7 m from the inside of the rear curb. The 0.15 m high brisket board was placed 1.8 m from the inside of the rear curb, which was 0.2 m high as measured from the alley floor. The concrete alleys were cleaned 8 times daily with an automated scraper; cross-over alleys were manually cleaned twice a day.

Cows were fed a Total Mixed Ration (TMR) formulated following the National Research Council (NRC) guidelines [17] to meet or exceed the requirements of a 658 kg Holstein producing 39 kg/d of milk with 4.6% fat; the TMR consisted of 30.3% corn silage, 42.4% concentrate mash, 5.9% grass haylage, 1.5% grass hay, 19.2% alfalfa hay and 0.7% wheat straw on a dry matter basis and was available *ad libitum* inside the barn. Fresh feed was provided at approximately 0700 and 1630 h. Feed was pushed up at approximately 1045, 1845 and 2230 h and feed refusals were removed at approximately 0530 h. In the experimental pen, cows had access to fresh water from a self-filling water trough located at the cross-over alley.

Cows were milked twice daily in a double-12 parallel milking parlor, between 0700 and 0800 h in the morning and between 1630 and 1730 h in the afternoon. Cows that were outside when they were collected for milking were moved directly from the outdoor pack to the parlor.

An automated selection gate allowed OUTDOOR cows free access to the open pack from the experimental pen. The outdoor open pack was 384 m^2^ covered by approximately 20 cm of sand which in turn was covered by approximately 25 cm of bark mulch. In addition, the pack contained 2 self-filling water troughs (approximately 16 m apart). Feces were removed every morning and afternoon when the cows were being milked.

### Group formation and training

Initially, 36 non-experimental (‘filler’) non-pregnant lactating cows were housed in the experimental pen. After enrolling the 60^th^ experimental cow, another 26 open filler cows were used to replace experimental cows at the end of their experimental period (i.e., > 84 DIM), to keep the group size consistent and group composition dynamic. At the start of the experiment, 18 filler cows were allowed access to the outdoor pack (OUTDOOR filler) and the other 18 were kept indoors (INDOOR filler). OUTDOOR cows (both filler and experimental cows) were trained to go through the selection gate that allowed them to go to the outdoor pack. INDOOR cows (both filler and experimental cows) were not trained; these cows could enter the selection gate but were redirected back to the indoor alley that led to the experimental pen (Fig 1). All cows were equipped with a neck-mounted sensor (Heatime, SCR Engineers) that was recognized by the selection gate and directed the cow relative to her assigned treatment.

Given that cows had no experience with using a selection gate and a one-way gate, experimental and filler OUTDOOR cows were trained over several sessions (at least 2 sessions) to use these gates in the experimental pen. The first session consisted of encouraging cows to approach the selection gate and to go through it, with all gates (i.e., sorting gate, and front and back gates of the automatic selection gate) in the open position, to access the indoor alley where they were rewarded with grain, fresh TMR and alfalfa hay. Cows were allowed access to the food rewards for approximately 1–2 min before being moved back to the pen through a one-way gate. The one-way gate was initially kept fully open, then half closed, and eventually fully closed for the final training sessions. Position of the one-way gate was changed to a more closed position once all cows of the group went through the one-way gates without hesitation. Each training session included a total of 4–5 loops through the selection gate; a maximum of 2 sessions occurred per day. When all cows met the learning criterion—i.e., went through the open selection gate without hesitation—the selection gate was then set on operating mode, in which the gates automatically opened and closed. At this point, cows were directed to the outdoor pack after entering the selection gate, where they could access the same food reward for approximately 3 min. Again, cows were required to complete 4–5 successful loops within one session. After the initial set of cows were trained, they were used to facilitate the training of naïve cows. Specifically, an experienced cow would enter the selection gate, after which the naïve cow would be encouraged to follow. Training was done in the experimental pen directly after the morning and afternoon milkings using groups of 3–5 naïve cows that had all calved within 9 d. Outside of training sessions (that all had a maximum duration of 1h), all cows could freely access the selection gate. In the days after training days, cows would be gently encouraged to enter the selection gate to go outside once daily, after afternoon milking, if they did not go outside on their own. Experimental cows met the learning criterion of going through the selection gate without a trainer present 6.2 ± 2.4 d (range: 1–10 d) after entering the experimental pen. On the rare occasion when cows for some reason stopped going outside after having met the training criteria, they were gently encouraged to enter the selection gate to go outside.

Once all filler cows were successfully trained and using the gate independently, the experiment started with experimental cows being enrolled into the experimental pen of fully trained filler cows (n = 36). Each time a cow calved, she was included in the experimental pen immediately following the first milking after calving, replacing a filler cow.

### Behavioral measures

Cow behavior was monitored continuously using video. Three dome video cameras (Panasonic WV-CW504SP, Sentinel Ultra-zoom w/Pan 1070 outdoor video camera, Sandpiper Technologies Inc., Manteca, CA) were attached at the outdoor wall of the barn at a height of 6 m to provide an overview of the outdoor pack. Additionally, 2 cameras were positioned 8 m above the experimental pen to provide an overview of the indoor lying area and 2 were positioned above the alley to provide an overview of this area and the selection gate. Three other cameras (Panasonic WVCP-470, Panasonic Corporation of North America, NJ, USA) were placed 6 m above the feed bunk to provide an overview of the feed bunk and alley. All video recordings were stored using a GeoVision 1480 digital recorder (USA Vision Systems, Irvine, CA). Red lights (BR38 red incandescent flood light, 100 W; Globe Electric Co. Inc., Montréal, QC, Canada) were placed adjacent to each camera to facilitate observation of cows at night. Each cow was dyed with a unique symbol on her back and sides to facilitate individual identification.

Cows wore a leg-mounted pedometer (AFI, AfiActII Pedometer Plus, Afimilk, Kibbutz Afikim, Israel), used to detect estrus. For the duration of all estrus periods (calculated as the number of hours the cow spent above the default threshold set by the manufacturer of the pedometer), each cow’s primary and secondary mounting behaviors (Table 1) were scored by continuous video observations. In addition, the location (i.e., the experimental pen, including the indoor alley, or the outdoor pack) where each behavior was performed was also recorded, as well as the time OUTDOOR cows spent inside and outside during their estrus. Monitoring for estrus events using the pedometer technology began directly after calving. Inter-observer reliability for each individual behavior was R^2^ > 0.89; inter-observer reliability for cow location was R^2^ = 1.0.

**Table 1:** Ethogram describing the estrus behaviors observed.

Behavior*	Definition
Standing to be mounted	The focal cow^1^ stands still whilst another cow mounts her from behind for at least 2 seconds^2^ (i.e., the front legs of the mounting cow will move in front of the cows’ Pelvic bones). The focal cow does not try to move away but may move a few steps over the duration of the mount to balance the weight of the other cow.
Mounting performed	The cow raises her body above that of another cow and clasps with her front legs in front of the other cows’ Pelvic bones. The cow that is being mounted may or may not walk away.
Attempted mount performed	The cow tries to mount another cow but is unsuccessful (i.e., the cow does not clasp her front legs in front of the other cows’ Pelvic bone) as the cow moves away forwards, sideways or backwards from her or because she is physically unable to.
Attempted mount received	The cow moves away forwards, sideways or backwards from another cow that is attempting to mount her or because the performing cow is physically unable to.
Disoriented mount performed	The cow raises her body above that of another cow and clasps with her front legs in any place other than the front of the other cows’ Pelvic bone.
Disoriented mount received	The cow is being mounted by another cow that clasps with her front legs in any place other than the front of the cows’ Pelvic bone.

* For each behavioral event the location where this event took place (i.e., inside the pen or on the outdoor pack) was noted. Ethogram is partially based on Palmer et al. [8].

^1^The focal cow is the cow whose behavior is observed.

^2^Following Sveberg et al. [3].

Following an alert from the pedometer, which was checked during morning and afternoon milking, the cow’s ovaries were examined by per rectum ultrasonography (Ibex Pro; E.I. Medical Imaging, Loveland, CO) to detect the presence of a dominant preovulatory follicle and to assess the presence or absence of a mature corpus luteum (CL). Per rectum ultrasonography was performed at the time of the alert as well as 7 d post-alert to confirm ovulation. Alerts were classified as true—and thus used within this study—when having at least one dominant pre-ovulatory follicle (greater than 15 mm) and an absence of a CL, or a CL that was less than 25 mm. Ovulation was confirmed at 7 d post-alert with the formation of a new CL. Rectal ultrasonography was done by locking the cow in a headlock within a sorting area that cows were automatically sorted into after milking, following the alert from the pedometer technology. When cows were not successfully sorted, rectal ultrasonography was conducted in the experimental pen by locking the cow in a headlock or by moving her into a pen fitted with freestalls. Events deemed as false alerts were not included in the study.

Experienced observers assessed the BCS during each estrus event. BCS was assessed using a 5-point scale (1 = thin, 5 = obese) with quarter point increments, following Edmonson et al. [18]. Cows were gait scored by experienced observers at every estrus event using a 5-point scale (1 = sound, 5 = severely lame; [14]).

### Climatic measures

Because our study lasted across several seasons, we included temperature and humidity index (THI) as a covariate. Air temperature and relative humidity inside the barn were recorded in 10-min intervals using a Hobo U23 Pro v2 Temperature/Relative Humidity Data Logger (Onset Computer Corp., Bourne, MA) placed at a height of 3 m in the middle of the experimental pen. Temperature-humidity index was calculated as: THI = (1.8T + 32)–[(0.55–0.0055 RH) x (1.8T – 26)] with T = air temperature (°C) and RH = relative humidity (%) [19].

### Statistical analyses

Due to severe winter weather we paused data collection from the 26^th^ of December 2017 until the 22^nd^ of January 2018; estrus events that occurred during this period were not assessed. During the remaining experimental period we recorded a total of 127 true estrus events. Thirty-three of these events were excluded from the analysis for the following reasons: during 6 events (2 OUTDOOR and 4 INDOOR cows) the pedometer system had technical issues; one INDOOR cow obtained outdoor access during her estrus; video analysis of all estrus events was completed by two observers, except for one estrus event for 4 OUTDOOR cows and one estrus event of an INDOOR cow, so we removed these 5 events to ensure high inter-observer reliability of all analyzed videos; 7 estrus events (of 4 OUTDOOR cows) were excluded as these cows were cycling irregularly. In another instance, an OUTDOOR cow obtained access to a non-experimental pen during her estrus event and in 4 instances, an INDOOR cow went through the selection gate and ended up in the indoor alley for several hours (as INDOOR cows were not trained to use the one-way gate that directed them back to the experimental pen); 3 OUTDOOR cows were excluded due to transponder failure (totalling 6 estrus events), causing them to be inconsistently denied access to the outdoors as they passed through the selection gate; one cow (INDOOR) died approximately 2 mo after calving and was replaced by a filler cow. The remaining 94 estrus events (46 of INDOOR cows; 48 of OUTDOOR cows) were used in the analyses.

Data were analyzed using SAS (version 9.4, SAS Institute, Institute Inc., Cary, NC), with cow as the experimental unit. The total number of estrous behaviors observed (Table 1) was calculated per estrus event per cow and expressed as frequency observed per hour of estrus (i.e., mounting intensity) in which cows were in the experimental pen (either indoor- or outdoors). Mounting intensity was used, as opposed to total number of mounts, to account for differences in estrus durations. All behaviors in Table 1 were summed together, as the different types of mounting behaviors were observed too infrequently to be analyzed separately. Model residuals of the pooled estrous behaviors were visually assessed for normality (which was attained) and outliers (which were not identified). A mixed model, using Type-1 Sum of Squares, was used to investigate the effect of treatment (i.e., INDOOR vs OUTDOOR) on mounting intensity and the duration of estrus. Cow was identified as subject, estrus event within cow was specified as a repeated measure, and an autoregressive Type 1 covariance structure was selected based on AIC. Models also assessed the following covariates (in this order): parity (i.e., primi- or multiparous), gait score (i.e., lame (gait score ≥3) or sound), 305ME, estrus event number (within cow), indoor THI, time of estrus onset as indicated by the pedometer system (classified as “day” 07:00–19:00, or “night” 19:00–07:00), and the total number of cows in estrus (i.e., no. of cows in true estrus at the same time as the cow of interest, as defined as at least 1 h overlap in their estrus events as indicated by the pedometer monitoring system). The latter covariate included estrus events of filler cows (including irregular cycling events) as well as the 29 removed estrus events. In addition, the interaction between each covariate and treatment was included. The final covariates and interactions included in each model were selected using backwards stepwise elimination. Trends (i.e., 0.1 < *P* > 0.05) were retained in all models. Significance was accepted at *P* < 0.05, and trends at *P* < 0.10.

## Results

### Descriptive statistics

A total of 3 OUTDOOR and 6 INDOOR cows failed to exhibit a true estrus event, as measured using the automated monitor. Of the cows that had at least 1 true estrus event, 7 INDOOR and 4 OUTDOOR cows never had a standing estrus. A total 8 INDOOR and 3 OUTDOOR cows did not show any of the scored estrous behaviors during at least 1 event; 2 of these INDOOR cows did not show any of the scored estrous behaviors during 2 estrus events. Average BCS of INDOOR and OUTDOOR cows was 2.9 ± 0.05 (range: 2.5–3.5) and 2.8 ± 0.05 (range: 2.5–3.25) respectively. OUTDOOR cows spent an average 1.6 ± 0.4 h outside when ≥ 1 INDOOR cow was in heat, compared to 1.3 ± 0.3 h when no INDOOR cows were in heat.

### Effect of treatment on mounting intensity

**Fig 2:**
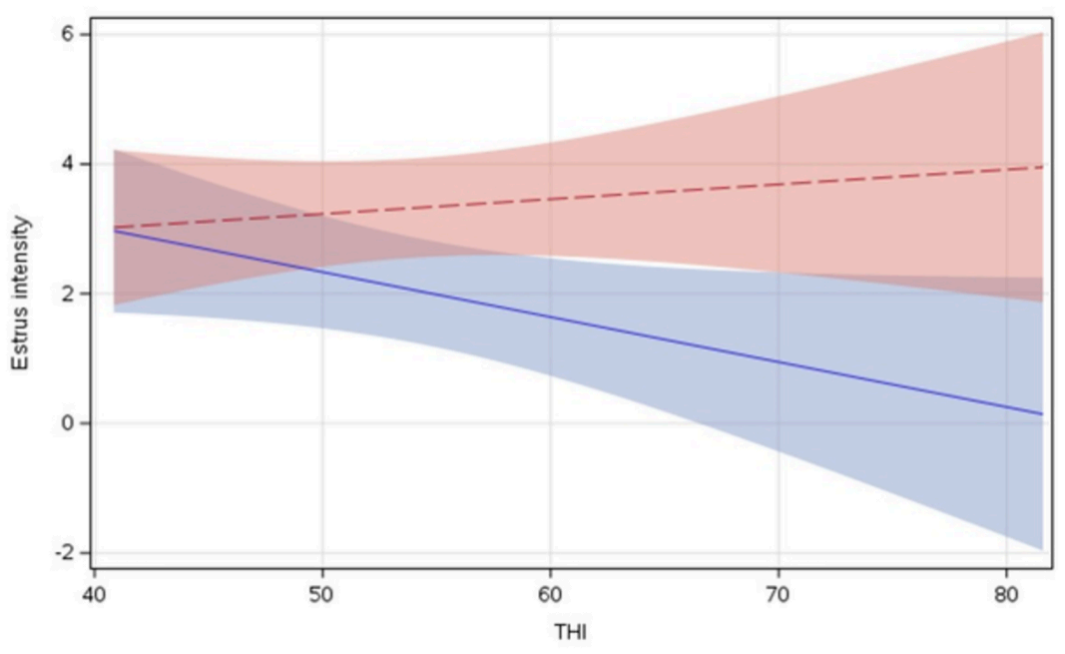
Relationship between indoor Temperature Humidity Index and estrus intensity, by treatment. OUTDOOR cows (red dashed line) versus INDOOR cows (blue solid line), including 95% confidence limits (slopes and CI from the model).

Access to an outdoor open wood-chip pack did not affect mounting intensity. The effect of treatment on mounting intensity tended to vary with THI (F_1,44_ = 3.68, *P* = 0.061); this interaction was driven by decreased mounting intensity of INDOOR cows with increasing indoor THI (Fig 2). The total number of cows in estrus (F_1,44_ = 24.36, *P* < 0.001) positively influenced mounting intensity. Estrus onset during the night (F_1,17_ = 3.18, *P* = 0.092) as well as estrus number (F_1,44_ = 3.07, *P* = 0.087) tended to be associated with a higher mounting intensity. Parity, gait score, and 305ME did not affect mounting intensity. Table 2 contains the descriptive statistics of the different estrous behaviors, separately for the 2 treatments.

**Table 2:** Descriptive statistics (mean (± SE)) regarding the number of estrous behaviors performed per hour during the duration of estrus as determined by an automated activity monitor for INDOOR (n = 21) and OUTDOOR (n = 24) that had at least 1 true estrus event and that did not have to be removed for other reasons.

	Treatment	
	INDOOR	OUTDOOR
Standing to be mounted	0.3 ± 0.1	0.5 ± 0.2
Mount performed	0.5 ± 0.1	1.0 ± 0.2
Attempted mount performed	0.3 ± 0.1	0.4 ± 0.1
Attempted mount received	0.1 ± 0.1	0.6 ± 0.1
Disoriented mount performed	0.1 ± 0.0	0.4 ± 0.1
Disoriented mount received	0.3 ± 0.1	0.3 ± 0.1
Mounting intensity (i.e., sum of all behaviours)	1.5 ± 0.3	3.2 ± 0.4

### Effect of treatment on estrus duration

OUTDOOR cows had a longer duration of estrus (mean ± SE: 12.4 ± 0.7 h) than did INDOOR cows (9.9 ± 0.8 h; F_1,43_ = 5.28, *P* = 0.027). In addition, estrus event number (F_1,47_ = 8.84, *P* = 0.005) and the total number of cows in estrus (F_1,47_ = 6.97, *P* = 0.011) were both positively associated with the duration of estrus. Parity, gait score, 305 ME, indoor THI and the time of estrus onset did not affect estrus duration.

## Discussion

We detected a tendency for an interaction between treatment and THI on mounting intensity, driven by INDOOR cows showing decreased mounting intensity with increasing indoor THI and no such relationship for OUTDOOR cows. These results for INDOOR cows are consistent with previous work showing that a period of warm weather decreases estrous intensity in cows [20]. Glucocorticoid concentrations tend to increase in cows experiencing heat stress, resulting in reduced duration of estrus [21]. Hansen et al. [22] postulated that cows experiencing heat stress lower their physical activity, including the expression of estrous behavior, to limit heat production.

OUTDOOR cows were able to access the outdoor pack, which potentially provided them with access to a cooler environment at night compared to the barn [23]. The ability of cows to select a cooler location may have helped them avoid the negative effects of heat stress. Overall lower indoor THI values at night likely explained why estrus intensity in both INDOOR and OUTDOOR cows tended to be higher with estrus onset during the night. The current study is, to our knowledge, the first to show that the benefits of outdoor access on mounting intensity are greater during periods of warmer weather. Heat stress in dairy cows is generally accepted to occur with THI values > 67 [24]. All estrus events in our study took place between August and February, with an average THI (± SD) of 55 ± 8.5 (ranging between: 40 and 82). The effect of an outdoor pack on mounting behavior may be stronger during periods of even warmer weather, but further work is needed to confirm this. Other studies have found a higher expression of estrous behaviors on pasture compared with a freestall barn [7, 8] and on outdoor dirt lots compared with an outdoor concrete floor [9, 13].

The number of cows in estrus influenced mounting intensity in our study, a finding also reported by Roelofs et al. [25]. Cows in estrus are more receptive to engaging in estrous behaviors [3]. Our study also indicated that estrus intensity tended to be longer with increasing estrus number (i.e., DIM), a result similar to Tippenhauer [26] who found that cows had greater odds of a high intensity estrus event as measured by peak activity change when DIM increased by 100 d.

OUTDOOR cows had a longer duration of estrus than INDOOR cows. The softer wood-chip flooring available on the outdoor pack may have increased the duration of estrus; this interpretation is consistent with Britt et al. [9] who found that the duration of estrus was longer when cows were observed on dirt compared with concrete. Estrus duration in our study was also influenced by estrus event number (i.e., higher DIM). In contrast, Stevenson et al. [27] detected no relationship between estrus duration and DIM. It is possible that individual cow characteristics, including the level of milk production, explain these differences: high milk production decreased estrus duration in a study by Lopez et al. [4].

We found that the duration of estrus increased with the number of cows in estrus, a result in line with previous work [28, 29]. Other studies have also found that the duration of estrus was influenced by factors such as season [30], BCS [31], lameness [32], parity [25] and milk production [4, 31].

Milk progesterone testing is a well-established method to detect true estrus events [33]. A cow with low intensity estrus behaviour may not have been detected by the activity monitoring system but could have been classified as being in true estrus via progesterone testing. However, given that increased activity was assessed based on each cows’ own activity baseline, this effect can be assumed to have been equal for INDOOR and OUTDOOR cows and would likely not have influenced our results.

Our study differed from previous work in that we allowed cows to choose between indoor and outdoor environments over multiple estrus events. Previous work did not provide cows a choice between different areas (e.g., [7, 8], or only let cows choose locations for a limited time [13]. Our study was also the first to investigate the effect of providing cows access to an outdoor pack on mounting intensity when both OUTDOOR and INDOOR cows were kept in the same pen by means of an automated selection gate. A strength of this approach is that all cows experienced the same physical and social indoor environment, allowing treatment to be applied at the cow level and decreasing the number of cows required for the study.

The study has several limitations. INDOOR cows may have shown less mounting intensity, due to not having access to OUTDOOR cows when these were on the outdoor pack, including when these animals were in estrus. OUTDOOR cows had more space to express estrus behaviours. OUTDOOR cows in heat spent more time outside when ≥ 1 INDOOR cow was in heat, perhaps reducing estrus intensity of both treatments. We only scored mounting during the estrus period, as indicated by the activity monitoring system based on elevated step-count; mounting behavior likely also occurred before and after this period. The use of positive reinforcement training may have motivated OUTDOOR cows to use the outdoor pack by creating a positive association with this environment. However, given that animals were tested over several weeks, we suggest that any effect of our initial training was likely to be minimal.

Visual estrus detection is the most used estrus detection method on Canadian dairy farms [34]; likely driven by the average herd size in Canada only being 73 cows [35]. The results of our study highlight that providing dairy cows continuous access to an outdoor area can help with visual estrus detection, perhaps improving the reproduction on dairy farms, but future studies are needed to investigate if access to an outdoor pack improves other reproduction indicators in dairy cattle.

## Conclusions

Increasing indoor THI tended to decrease mounting intensity of INDOOR cows, but not for OUTDOOR cows. OUTDOOR cows had a longer duration of estrus than INDOOR cows. We conclude that access to an outdoor open pack facilitates estrus behavior in freestall housed dairy cows, especially during times of higher THI.

## References

[1] Diskin MGD, Sreenan JMS. Expression and detection of oestrus in cattle. Reprod Nutr Dev. 2000;40:481–91. doi: 10.1051/rnd:2000112 11140818

[2] Forde N, Beltman ME, Lonergan P, Diskin M, Roche JF, Crowe MA. Oestrous cycles in Bos taurus cattle. Anim Reprod Sci. 2011;124(3–4):163–9. doi: 10.1016/j.anireprosci.2010.08.025 20875708

[3] Sveberg G, Refsdal AO, Erhard HW, Kommisrud E, Aldrin M, Tvete IF, et al. Behavior of lactating Holstein-Friesian cows during spontaneous cycles of estrus. J Dairy Sci. 2011;94(3):1289–301. doi: 10.3168/jds.2010-3570 21338794

[4] Lopez H, Satter LD, Wiltbank MC. Relationship between level of milk production and estrous behavior of lactating dairy cows. Anim Reprod Sci. 2004;81:209–23. doi: 10.1016/j.anireprosci.2003.10.009 14998648

[5] Saint-Dizier M, Chastant-Maillard S. Towards an automated detection of oestrus in dairy cattle. Reprod Domest Anim. 2012 Dec;47(6):1056–61. doi: 10.1111/j.1439-0531.2011.01971.x 22214367

[6] Denis-Robichaud J, Cerri RLA, Jones-Bitton A, LeBlanc SJ. Dairy producers’ attitudes toward reproductive management and performance on Canadian dairy farms. J Dairy Sci. 2018;101(1):850–60. doi: 10.3168/jds.2016-12416 29055532

[7] Palmer MA, Olmos G, Boyle LA, Mee JF. Estrus detection and estrus characteristics in housed and pastured Holstein-Friesian cows. Theriogenology. 2010;74(2):255–64. doi: 10.1016/j.theriogenology.2010.02.009 20451993

[8] Palmer MA, Olmos G, Boyle LA, Mee JF. A comparison of the estrous behavior of Holstein-Friesian cows when cubicle-housed and at pasture. Theriogenology. 2012;77(2):382–8. doi: 10.1016/j.theriogenology.2011.08.010 21924470

[9] Britt JH, Scott RG, Armstrong JD, Whitacre MD. Determinants of Estrous Behavior in Lactating Holstein Cows. J Dairy Sci. 1986;69(8):2195–202. doi: 10.3168/jds.S0022-0302(86)80653-1 3760306

[10] Telezhenko E, Bergsten C. Influence of floor type on the locomotion of dairy cows. Appl Anim Behav Sci. 2005;93:183–97.

[11] van der Tol PPJ, Metz JHM, Back W, Braam CR, Weijs WA. Frictional Forces Required for Unrestrained Locomotion in Dairy Cattle. J Dairy Sci. 2005;88(2):615–24. doi: 10.3168/jds.S0022-0302(05)72725-9 15653528

[12] Boyle LA, Boyle RM, French P. Welfare and performance of yearling dairy heifers out-wintered on a woodchip pad or housed indoors on two levels of nutrition. Animal. 2008;2(5):769–78. doi: 10.1017/S1751731108001870 22443603

[13] Vailes LD, Britt JH. Influence of footing surface on mounting and other sexual behaviors of estrual Holstein cows. J Anim Sci. 1990;68(8):2333–9. doi: 10.2527/1990.6882333x 2401655

[14] Flower FC, Weary DM. Effect of hoof pathologies on subjective assessments of dairy cow gait. J Dairy Sci. 2006;89(1):139–46. doi: 10.3168/jds.S0022-0302(06)72077-X 16357276

[15] Silvia WJ, Hatler TB, Nugent AM, Laranja Da Fonseca LF. Ovarian follicular cysts in dairy cows: An abnormality in folliculogenesis. Domest Anim Endocrinol. 2002;23(1–2):167–77. doi: 10.1016/s0739-7240(02)00154-6 12142235

[16] Jeengar K, Chaudhary V, Kumar A, Raiya S, Gaur M, Purohit GN. Ovarian cysts in dairy cows: Old and new concepts for definition, diagnosis and therapy. Anim Reprod. 2014;11(2):63–73.

[17] NRC. Nutrient Requirements of Dairy Cattle. 7th rev. e. Nutrient Requirements of Dairy Cattle. Washington, DC: National Academic Press; 2001. 401 p.

[18] Edmonson AJ, Lean IJ, Weaver LD, Farver T, Webster G. A Body Condition Scoring Chart for Holstein Dairy Cows. J Dairy Sci. 1989;72(1):68–78. Available from: http://linkinghub.elsevier.com/retrieve/pii/S0022030289790810

[19] Ravagnolo O, Misztal I, Hoogenboom G. Genetic Component of Heat Stress in Dairy Cattle, Development of Heat Index Function. J Dairy Sci. 2000;83(9):2120–5. doi: 10.3168/jds.S0022-0302(00)75094-6 11003246

[20] Orihuela Â. Some factors affecting the behavioural manifestation of oestrus in cattle: a review. Appl Anim Behav Sci. 2000;70:1–16. doi: 10.1016/s0168-1591(00)00139-8 10986419

[21] Hein KG, Allrich RD. Influence of exogenous adrenocorticotropic hormone on estrous behavior in cattle. J Anim Sci. 1992;70(1):243–7. doi: 10.2527/1992.701243x 1316341

[22] Hansen PJ, Drost M, Rivera RM, Paula-Lopes FF, Al-Katanani YM, Krininger CE, et al. Adverse impact of heat stress on embryo production: Causes and strategies for mitigation. Theriogenology. 2001;55(1):91–103. doi: 10.1016/s0093-691x(00)00448-9 11198091

[23] VanderZaag A, Le Riche E, Baldé H, Kallil S, Ouellet V, Charbonneau É, et al. Comparing thermal conditions inside and outside lactating dairy cattle barns in Canada. J Dairy Sci. 2023;106(7):4738–58. doi: 10.3168/jds.2022-22870 37225574

[24] Polsky L, von Keyserlingk MAG. Invited review: Effects of heat stress on dairy cattle welfare. J Dairy Sci. 2017;100(11):8645–57. doi: 10.3168/jds.2017-12651 28918147

[25] Roelofs JB, Van Eerdenburg FJCM, Soede NM, Kemp B. Various behavioral signs of estrous and their relationship with time of ovulation in dairy cattle. 2005;63:1366–77. 10.1016/j.theriogenology.2004.07.009 15725444

[26] Tippenhauer CM, Plenio JL, Madureira AML, Cerri RLA, Heuwieser W, Borchardt S. Factors associated with estrous expression and subsequent fertility in lactating dairy cows using automated activity monitoring. J Dairy Sci. 2021;104(5):6267–82. doi: 10.3168/jds.2020-19578 33663844

[27] Stevenson JS, Hill SL, Nebel RL, Dejarnette JM. Ovulation timing and conception risk after automated activity monitoring in lactating dairy cows. J Dairy Sci. 2014;97(7):4296–308. doi: 10.3168/jds.2013-7873 24819138

[28] Hurnik JF, King GJ, Robertson HA. Estrous and related behaviour in postpartum Holstein cows. Appl Anim Ethol. 1975;2:55–68.

[29] Van Vliet JH, Van Eerdenburg FJCM. Sexual activities and oestrus detection in lactating Holstein cows. Appl Anim Behav Sci. 1996;50(1):57–69.

[30] De Rensis F, Scaramuzzi RJ. Heat stress and seasonal effects on reproduction in the dairy cow—A review. Theriogenology. 2003;60(6):1139–51. doi: 10.1016/s0093-691x(03)00126-2 12935853

[31] Madureira AML, Silper BF, Burnett TA, Polsky L, Cruppe LH, Veira DM. Factors affecting expression of estrus measured by activity monitors and conception risk of lactating dairy cows. J Dairy Sci. 2015;98(10):7003–14. doi: 10.3168/jds.2015-9672 26254517

[32] Walker SL, Smith RF, Routly JE, Jones DN, Morris MJ, Dobson H. Lameness, Activity Time-Budgets, and Estrus Expression in Dairy Cattle. J Dairy Sci. 2008;91(12):4552–9. doi: 10.3168/jds.2008-1048 19038930 PMC2729911

[33] Friggens NC, Bjerring M, Ridder C, Højsgaard S, Larsen T. Improved Detection of Reproductive Status in Dairy Cows Using Milk Progesterone Measurements. Reprod Domest Anim. 2008;43(SUPPL.2):113–21. doi: 10.1111/j.1439-0531.2008.01150.x 18638112

[34] Van Schyndel SJ, Bauman CA, Pascottini OB, Renaud DL, Dubuc J, Kelton DF. Reproductive management practices on dairy farms: The Canadian National Dairy Study 2015. J Dairy Sci. 2019;102(2):1822–31. doi: 10.3168/jds.2018-14683 30594369

[35] Luby CD, Waldner C, Jelinski MD. Update on demographics of the Canadian Dairy Industry for the period 2011 to 2016. Can Vet J. 2020;61:75–8. 31892759 PMC6909404

